# Supplementary material for: Feature Selection based on the Local Lift Dependence Scale
Source: Entropy (Basel). 2018 Jan 30;20(2):97. doi: 10.3390/e20020097 (PMC7512664; doi:10.3390/e20020097)
Supplement: Supplementary file 1 [file entropy-20-00097-s001.zip › Manual localift.pdf]

Package ‘localift’

November 11, 2017

Type Package

Title Functions to manipulate data in order to study the Local Lift Dependence (Beta Version)

Version 0.0.1

URL http://github.com/dmarcondes/localift

BugReports https://github.com/dmarcondes/localift/issues

Description Generates tables, plots and measures related to the Local Lift Dependence.

Imports ggplot2 (>= 2.2.1), MASS (>= 7.3-45),

Depends R (>= 3.3.2)

License GPL-3

Encoding UTF-8

LazyData true

RoxygenNote 6.0.1

R topics documented:

heatmap.lift 2
lift.feat.selection 3
LiftTable 5
mahalanobis.plot 8
requirement 9

Index **11**

heatmap.lift *Lift Function Heatmap*

Description

Heatmap of the Lift Function.

Usage

```
heatmap.lift(x, y, xlab = "x", ylab = "y", plot.title = " ",
  palette = "Spectral", na.color = "red", contour = TRUE,
  contour.color = "white", contour.alpha = 0.5, contour.bin = 15,
  contour.binwidth = 1, critical.value = 3, quantilesx = c(1/3, 2/3),
  quantilesy = c(1/3, 2/3), breaks = c(1, 2), quantile.type = 7,
  n = length(x), grid = TRUE)
```

Arguments

x	A vector of data.
y	A vector of data.
xlab	Label of the x-axis.
ylab	Label of the y-axis.
plot.title	Title of the heatmap plot.
palette	The color palette to be used on the heatmap. Default is *'Spectral'*.
na.color	Color for *NA* values.
contour	Logical. Whether to plot the Lift Function contour.
contour.color	Color of the Lift Function contour.
contour.alpha	Transparency coefficient for the contour plot.
contour.bin	Number of bins for the contour plot.
contour.binwidth	Width of the bins for the contour plot.
critical.value	Critical value in which the Lift Function must be truncated.
quantilesx	Quantiles to be marked on the x-axis.
quantilesy	Quantiles to be marked on the y-axis.
breaks	Breaks of the heatmap legend. Must not contain *0* nor *critical.value* and all values must be less than the *critical.value*.
quantile.type	An integer between 1 and 9 selecting one of the nine quantile algorithms of the *quantile* function.
n	Number of equally spaced points at which the density is to be estimated. When n > 512, it is rounded up to a power of 2 during the calculations (as fft is used) and the final result is interpolated by approx. So it almost always makes sense to specify n as a power of two
grid	Logical. Whether to plot grid lines representing the sample quantiles.

Details

Given two continuous random variables returns the heatmap of their estimated Lift Function. The heatmap paint the support of *(x,y)* according to the values of the Lift Function $L(x,y) = f(x,y)/(g(x)*h(y))$ in which *f(x,y)* is the estimated joint density of *(x,y)* and *g(x)* and *h(y)* are the respective estimated marginal distributions. If $L(x,y) > critical.value$ then *L(x,y) = critical.value*, i.e., *L(x,y)* is truncated at *critical.value*.

Value

`plot`	A ggplot2 heatmap.

References

Simonis, A., Marcondes, D., Barrera, J. (2017) Feature Selection based on the Local Lift Dependence Scale. *Submitted*

Examples

```
attach(requirement)
heatmap.lift(SP,P2)
```

`lift.feat.selection` *Lift Featuring Selection Algorithm*

Description

Given two sets *x* and *y*, select the subsets of variables that are more dependent.

Usage

```
lift.feat.selection(y, x, block.x = NULL, block.y = NULL, fix.x = NULL,
  fix.y = NULL, reach.x = "all", reach.y = "all", cost = "eta",
  local = NULL, low.min = 0, windows = "all", trace = FALSE,
  se = FALSE, quantilesx = c(1/3, 2/3), quantile.type = 7,
  namesx = c("1 TertileX", "2 TertileX", "3 TertileX"), quantilesy = c(1/3,
  2/3), namesy = c("1 TertileY", "2 TertileY", "3 TertileY"),
  method = "mahalanobis", p.minkowski = 1, sample = "se",
  point = "zero", min.size = 0, right = TRUE)
```

Arguments

`y`	Data frame in which each column is a sample of a random variable.
`x`	Data frame in which each column is a sample of a random variable.
`block.x`	Block factor in which x must be discretize within. The quantiles will be taken inside each block.

`block.y`	Block factor in which y must be discretize within. The quantiles will be taken inside each block.
`fix.x`	Number of the columns of x that must be considered in all iterations.
`fix.y`	Number of the columns of y that must be considered in all iterations.
`reach.x`	Vector with the sizes of the subsets of x that must be considered. Default is all the power set of x.
`reach.y`	Vector with the sizes of the subsets of y that must be considered. Default is all the power set of y.
`cost`	Cost function to be used. Must be *"MI"* for Mutual Information, *"eta"* for *eta* on windows or 'max' for the local max.
`local`	If *cost = 'max'*, must be the name of the category of *y* which the maximum of the lift must be searched for. Must be a level of *y* or one of the labels in *namesy*.
`low.min`	If *cost = 'max'*, must be an lower bound for the profile relative frequency, so that the algorithm will search for the rows of the lift table with relative frequency greater than *low.min*.
`windows`	In what windows the eta coefficient must be calculated. Can be *"all"* for all windows or *"cat"* for the windows that are given by a single category od *U*.
`trace`	Logical. Whether to return a data frame with the mutual information and the eta coefficient, along with the Lift Table, for every subset of *y* and *x*.
`se`	Whether the standard error must be calculated for the lift table and the eta coefficients. If *TRUE* the computational time increases exponentially.
`quantilesx`	Quantiles in which the variables *x* must be partitioned. Must not contain *0* nor *1*.
`quantile.type`	An integer between 1 and 9 selecting one of the nine quantile algorithms of the *quantile* function.
`namesx`	Name of the partitions of *x*. Its length must equal *length(quantilesx) + 1*.
`quantilesy`	Quantiles in which the variables *y* must be partitioned. Must not contain *0* nor *1*.
`namesy`	Name of the partitions of *y*. Its length must equal *length(quantilesy) + 1*.
`method`	Method that should be used to calculate the distance between the sample points and zero or the sample mean. Must be *'mahalanobis'*, *'euclidean'*, *'maximum'*, *'manhattan'*,*'canberra'*, *'binary'* or *'minkowski'*.
`p.minkowski`	The power of the Minkowski distance, if *method = 'minkowski'*.
`sample`	What information about the sample to display in parenthesis on the Lift Table. Must *'size'* for sample size, *'joint'* for relative frequency and *'none'* for displaying nothing.
`point`	Which point to calculate the distance from. Must be *'zero'* or *'mean'* for sample mean.
`min.size`	Minimum sample size that can be considered. It will skip all combinations of the *x* and *y* partitions in which the sample size is less than *min.size*
`right`	Logical, indicating if the quantile intervals should be closed on the right (and open on the left) or vice versa.

Details

Given two sets *x* and *y* of random variables, it returns the subsets *x'* of *x* and *y'* of *x* for which the Global Eta Coefficient, or W-Local Eta Coefficient, or the Lift Function at a given point is the greatest in comparison to all the other combination of subsets of *x* and *y*.

Value

`lift`	The LiftTable of the selected features.
`MI`	The Mutual information between the returned subsets.
`Eta`	The dependence measures eta between the returned subsets.
`EtaM`	The maximum eta dependence measure in windows of the returned x subset.
`Window`	The window with maximum eta coefficient.
`Max`	The maximum of the lift on the considered category of *y*.
`LevelMax`	The levels of *x* for which the lift is *max*.
`subsetx`	The subset of *x*
`subsety`	The subset of *y*
`xquantile`	The quantile of each observation of *x*.
`yquantile`	The quantile of each observation of *y*.
`trace`	The table with the trace of the algorithm.
`ltables`	The Lift Tables of each iteration.

References

Simonis, A., Marcondes, D., Barrera, J. (2017) Feature Selection based on the Local Lift Dependence Scale. *Submitted*

Examples

```
attach(requirement)
lift.feat.selection(SP,data.frame(C1,C2,C3,P1,P2),cost = "eta",trace = TRUE,local = "3 TertileY")
```

`LiftTable` *Lift Table*

Description

Lift Table between two groups of variables.

Usage

```
LiftTable(table = NULL, x, y, se = TRUE, local = NULL, w = TRUE,
  windows = "all", block.x = 1, block.y = 1, quantilesx = c(1/3, 2/3),
  namesx = c("1 TertileX", "2 TertileX", "3 TertileX"), quantilesy = c(1/3,
  2/3), namesy = c("1 TertileY", "2 TertileY", "3 TertileY"),
  quantile.type = 7, point.x = "zero", point.y = "zero",
  method = "mahalanobis", p.minkowski = 1, low.min = 0, sample = "se",
  right = TRUE, digits = 3)
```

Arguments

`table`	A contingency table without margins and with row and column names. Needed only if the factor variables are already tabulated.
`x`	A numeric matrix, a factor vector or a numeric vector.
`y`	A numeric matrix, a factor vector or a numeric vector.
`se`	Whether the standard error must be calculated for the lift table and the eta coefficients.
`local`	Must be the name of the category of *y* in which the maximum of the lift must be searched for. Must be a level of *y* or one of the labels in *namesy*. The *default* is the first category of *y*.
`w`	Whether the *eta* coefficient must be calculated for the *windows* of the support of *U*.
`windows`	In what windows the eta coefficient must be calculated. Must be a list of vectors, in which each vector contain the name of the categories of *U*. Can be *"all"* for all windows or *"cat"* for the windows that are given by a single category od *U*
`block.x`	Block factor in which x must be discretize within. The quantiles will be taken inside each block.
`block.y`	Block factor in which y must be discretize within. The quantiles will be taken inside each block.
`quantilesx`	Quantiles in which the variable *x* must be partitioned. Must not contain *0* nor *1*.
`namesx`	Name of the partitions of *x*. Its length must equal *length(quantilesx) + 1*.
`quantilesy`	Quantiles in which the variables *y* must be partitioned. Must not contain *0* nor *1*.
`namesy`	Name of the partitions of *y*. Its length must equal *length(quantilesy) + 1*.
`quantile.type`	An integer between 1 and 9 selecting one of the nine quantile algorithms of the quantile function.
`point.x`	Which point to calculate the distances of x from. Must be *'zero'*, *'mean'* for sample mean or a vector with the dimension of x.
`point.y`	Which point to calculate the distances of y from. Must be *'zero'*, *'mean'* for sample mean or a vector with the dimension of y.
`method`	Method that should be used to calculate the distance. Must be *'mahalanobis'*, *'euclidean'*, *'maximum'*, *'manhattan'*,*'canberra'*, *'binary'* or *'minkowski'*.
`p.minkowski`	The power of the Minkowski distance, if *method = 'minkowski'*.

`low.min`	If *cost = 'max'*, must be an lower bound for the profile relative frequency, so that the algorithm will search for the rows of the lift table with relative frequency greater than *low.min*.
`sample`	What information about the sample to display in parenthesis on the Lift Table. Must be *'size'* for sample size, *'joint'* for relative frequency, *'se'* for standard error and *'none'* for displaying nothing.
`right`	Logical, indicating if the quantile intervals should be closed on the right (and open on the left) or vice versa.
`digits`	Integer indicating the number of significant digits (signif) to be used on the Lift Table.

Details

If *x* are *y* are numeric vectors, it computes the Lift Table between the discrete random variables *U* and *V* determined from the sample quantiles of *x* and *y*, inside each level of the block variables. If *x* or *y* are numerical *matrices*, then the Lift Table is computed from the discrete random variables *U* and *V* determined from the partition of *(x,y)* by the selected distance method based on the sample quantiles, within each level of the block factors. If *x* or *y* are factor vectors, the Lift Function is calculated directly from *x* and *y*. If *x* or *y* contain numerical and factor variables then the discretization process is made by the quantile discretization of the sample distances from the given point, within each level of the factor and block variables.

If *(U,V)* are discrete and *(u,v)* is a category of *(U,V)* then the Lift Function at such category is given by $L(u,v) = p(u,v)/(pU(u) * pV(v))$ in which *p* is the relative joint frequency of *(U,V)* and *pU* and *pV* are the respective marginal relative frequencies. For more details see the References.

Value

`ltable`	The Lift Table. Rows are related to *x* and columns to *y*.
`eta`	The dependence measures eta.
`MI`	The normalized Mutual Information of the Lift Table.
`max`	The maximum of the lift on the considered category of *y*.
`xquantile`	The quantile of each observation of *x*.
`yquantile`	The quantile of each observation of *y*.
`se`	The standard error for each lift value.
`cov`	The covariance matrix of the lift function in lexicographical order.

References

Simonis, A., Marcondes, D., Barrera, J. (2017) Feature Selection based on the Local Lift Dependence Scale. *Submitted*

Examples

```
attach(requirement)
LiftTable(x = SP,y = data.frame(C3,P1))
LiftTable(x = data.frame(C1,C2,C3),y = data.frame(P1,P2))
LiftTable(x = data.frame(iris[,1:4]),y = iris$Species)
```

mahalanobis.plot *Mahalanobis Plot*

Description

Dispersion plot with the Mahalanobis partition ellipses.

Usage

```
mahalanobis.plot(x, y, g = NULL, g.color = "black", g.name = "Group",
  xlab = "x", ylab = "y", xlim = c(min(data[, 1]), max(data[, 1])),
  ylim = c(min(data[, 2]), max(data[, 2])), quantiles = c(1/3, 2/3),
  names = c("1 Tertile", "2 Tertile", "3 Tertile"), partition = TRUE,
  rectangle = TRUE, rectangle.color = "black", plot.title = " ",
  point = "zero", quantile.type = 7, shape = 16, size = 1.5,
  color = "black")
```

Arguments

x	A vector of data.
y	A vector of data.
g	Grouping factor.
g.color	A vector with color names for the ellipses. It must present one color for each level of the grouping factor.
g.name	Name of the grouping factor.
xlab	Label of the x-axis.
ylab	Label of the y-axis.
xlim	Limits of the x-axis.
ylim	Limits of the y-axis.
quantiles	Quantiles of the Mahalanobis partition.
names	Name of the partitions. Its length must equal *length(quantiles) + 1*.
partition	Logical. Whether the Mahalanobis ellipses are plotted.
rectangle	Logical. Whether a rectangle is plotted around the data.
rectangle.color	Color of the rectangle.
plot.title	Title of the plot.
point	Which point to calculate the distance from. Must be *'zero'* or *'mean'* for sample mean.
quantile.type	Integer between 1 and 9 selecting one of the nine quantile algorithms of the *quantile* function.
shape	ggplot2 shape.
size	ggplot2 size.
color	Points point color.

Details

Dispersion plot of two random variables with the ellipses of the Mahalanobis partition given by the quantiles of a distances to a given point inside each level of a grouping factor.

Value

`plot`	The dispersion plot.
`quantiles`	The quantile of each observation of *data*.

References

Simonis, A., Marcondes, D., Barrera, J. (2017) Feature Selection based on the Local Lift Dependence Scale. *Submitted*

Examples

```
attach(requirement)
mahalanobis.plot(C1,C2,xlim = c(0,10),ylim = c(0,10))
```

`requirement` *Requirement data set*

Description

Data set containing the performance of 61 undergraduate Statistics students in Stochastic Process and its required courses.

Usage

```
requirement
```

Format

An object of class `data.frame` with 61 rows and 8 columns.

Details

The performance in each course is taken as the grade in the course, a number between *0* and *10*.

Value

`C1`	The performance in Calculus I.
`C2`	The performance in Calculus II.
`C3`	The performance in Calculus III.
`P1`	The performance in Probability I.
`P2`	The performance in Probability II.
`SP`	Performance in Stochastic Process.
`Year`	The year each student enrolled in the undergraduate course.

References

Simonis, A., Marcondes, D., Barrera, J. (2017) Feature Selection based on the Local Lift Dependence Scale. *Submitted*

Index

*Topic **datasets**
    requirement, 9

heatmap.lift, 2

lift.feat.selection, 3
LiftTable, *5*, 5

mahalanobis.plot, 8

quantile, *6*

requirement, 9
